# Supplementary material for: Identification of photoperiod-induced specific miRNAs in the adrenal glands of Sunite sheep (Ovis aries)
Source: Front Vet Sci. 2022 Jul 22;9:888207. doi: 10.3389/fvets.2022.888207 (PMC9354845; doi:10.3389/fvets.2022.888207)
Supplement: Supplementary file 1 [file Data_Sheet_1.ZIP › Supplementary file/miRDeep2 file.zip!/miRDeep2 file/Know_Structure/SPLP42A3/oar-mir-329a.pdf]

miRBase precursor : oar-mir-329a
Total read count : 45
oar-miR-329a-5p read count : 5
oar-miR-329a-3p read count : 39
remaining reads : 1

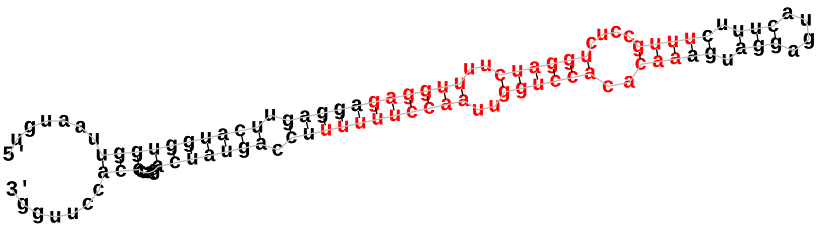

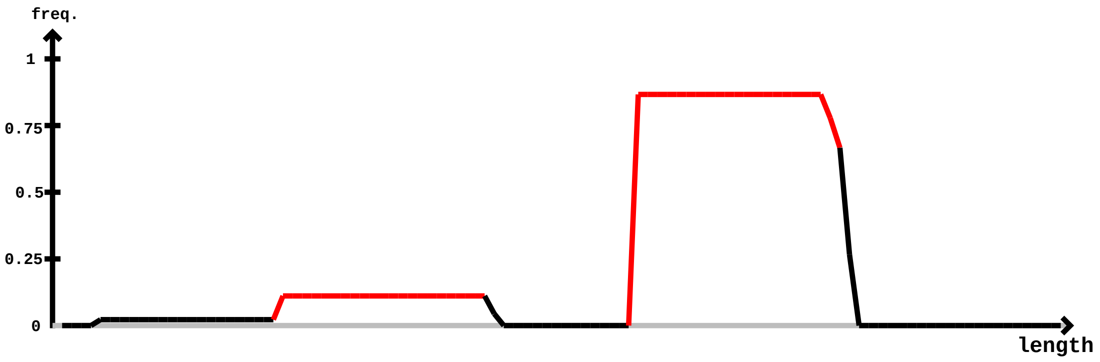


```
                      oar-miR-329a-5p
                                                    oar-miR-329a-3p
5'- uguaauuggugguacuugaggagagguuuucuaggucuccguuucuuucaugaggaugaaacacaccugguuaaccuuuuuuccaguaucagaucccaccuugg  -3'  exp
    .......(((((((((.(((((((((((..((((((....(((((.(((....))).)))))..))))))..))))))))))).)))).......))))).... reads  mm  sample
    ...aauuggugguacuugagga.................................................................................. 1      0   seq
    ......................gagguuuucuaggucuccguuu............................................................ 3      0   seq
    ......................gagguuuucuaggucuccguuuc........................................................... 2      0   seq
    ..........................................................aacacaccugguuaaccuuu......................... 4      0   seq
    ..........................................................aacacaccugguuaaccuuuu........................ 5      0   seq
    ..........................................................aacacaccugguuaaccuuuuu....................... 18     0   seq
    ..........................................................aacacaccugguuaaccuuuuuu...................... 12     0   seq
```
